# Genome-wide association reveals genetic variation of lint yield components under salty field conditions in cotton (*Gossypium hirsutum* L.)

**DOI:** 10.1186/s12870-019-2187-y

**Published:** 2020-01-14

**Authors:** Guozhong Zhu, Wenwei Gao, Xiaohui Song, Fenglei Sun, Sen Hou, Na Liu, Yajie Huang, Dayong Zhang, Zhiyong Ni, Quanjia Chen, Wangzhen Guo

**Affiliations:** 1 State Key Laboratory of Crop Genetics and Germplasm Enhancement, Engineering Research Center of Hybrid Cotton Development (the Ministry of Education), Nanjing Agricultural University, Nanjing, 210095 China; 2 Engineering Research Center for Cotton (the Ministry of Education), Xinjiang Agricultural University, Urumqi, 830052 China

**Keywords:** Genome-wide association study, Lint percentage, Boll number per plant, Boll weight per plant, Salt stress, Transcriptome analysis, *Gossypium hirsutum*

## Abstract

**Background:**

Salinity is one of the most significant environmental factors limiting the productivity of cotton. However, the key genetic components responsible for the reduction in cotton yield in saline-alkali soils are still unclear.

**Results:**

Here, we evaluated three main components of lint yield, single boll weight (SBW), lint percentage (LP) and boll number per plant (BNPP), across 316 *G. hirsutum* accessions under four salt conditions over two years. Phenotypic analysis indicated that LP was unchanged under different salt conditions, however BNPP decreased significantly and SBW increased slightly under high salt conditions. Based on 57,413 high-quality single nucleotide polymorphisms (SNPs) and genome-wide association study (GWAS) analysis, a total of 42, 91 and 25 stable quantitative trait loci (QTLs) were identified for SBW, LP and BNPP, respectively. Phenotypic and QTL analysis suggested that there was little correlation among the three traits. For LP, 8 stable QTLs were detected simultaneously in four different salt conditions, while fewer repeated QTLs for SBW or BNPP were identified. Gene Ontology (GO) analysis indicated that their regulatory mechanisms were also quite different. Via transcriptome profile data, we detected that 10 genes from the 8 stable LP QTLs were predominantly expressed during fiber development. Further, haplotype analyses found that a MYB gene (*GhMYB103*), with the two SNP variations in cis-regulatory and coding regions, was significantly correlated with lint percentage, implying a crucial role in lint yield. We also identified that 40 candidate genes from BNPP QTLs were salt-inducible. Genes related to carbohydrate metabolism and cell structure maintenance were rich in plants grown in high salt conditions, while genes related to ion transport were active in plants grown in low salt conditions, implying different regulatory mechanisms for BNPP at high and low salt conditions.

**Conclusions:**

This study provides a foundation for elucidating cotton salt tolerance mechanisms and contributes gene resources for developing upland cotton varieties with high yields and salt stress tolerance.

## Background

Salinity is one of the most significant environmental factors limiting the productivity of crop plants [1]. Salinity stress affects about one billion hectares of arid and semi-arid areas globally [2] and is becoming progressively more severe due to climatic changes, unscientific irrigation and excessive fertilization [3]. Scientists and agronomists have expended much effort to make improvements and increase utilization of saline soil. In addition to using physical and chemical methods to reduce salt content, screening or breeding high-salt-tolerant crops by modern molecular means is an economic and effective way to solve the present situation.

In the past few decades, several methods, such as molecular markers [4], transgene technology [5], transcriptome sequencing [6], and genome-wide association study (GWAS) based on single nucleotide polymorphism (SNP) [7], have been used for investigating the mechanism of salt tolerance and for mining elite alleles in plants. GWAS is used widely in plants because of its ability to effectively associate genotypes with phenotypes and detect many natural allelic variations simultaneously using natural populations. In rice, Shi et al. (2017) reported 11 loci associated with stress-susceptibility indices (SSIs) of vigor index (VI) and mean germination time (MGT) by screening 6,361,920 SNPs on 478 accessions, and this has potential value in future molecular assisted breeding to improve stress tolerance [8]. Cotton (*Gossypium spp.*) could be used for soil reclamation as a pioneer crop of saline-alkali land due to its high salt tolerance [9]. However, its growth and development can still be affected by adverse salt conditions. Soil salinity ranging from 8 to 18 dS/m resulted in yield losses of 15 to 55% in cotton [10]. Therefore, discovering the limiting factors and identifying the genes involved in the salt-tolerance response pathway is an effective way to increase cotton yield.

Many candidate genes that underlie traits such as cotton fiber yield and quality [11–14], and seed oil composition and protein content [15], have been revealed. In addition, several studies on stress tolerance have been carried out in cotton. Using 145 simple-sequence repeat (SSR) markers, 60 QTLs associated with ten salt-tolerance related traits were detected [4]. Through GWAS and RNA-seq analysis, 33 significant SNPs associated with comprehensive evaluation values of salt tolerance were identified in cotton [9]. Via genome-wide SNP analysis through genotyping by sequencing (GBS), a total of 66 QTLs for 10 traits related to salinity were identified, and 12 candidate genes in these QTLs might play crucial roles in salt tolerance in cotton [16]. Using a CottonSNP63K array, a total of 23 SNPs that represented seven genomic regions were significantly associated with two salt-tolerance-related traits, relative survival rate and salt tolerance level, at the seedling stage in upland cotton [7]. Using genome re-sequencing data, nine SNP rich regions associated with relative fresh weight, relative stem length, relative water content and comprehensive index of salt tolerance under salt conditions were reported in Asiatic cotton [17]. Several studies have focused on salt-tolerance related traits, however, few reports have investigated fiber yield under stress conditions such as salt stress, despite this having more practical value for saline-alkali soil utilization.

In the current study, we investigated three main components of lint yield, single boll weight (SBW), lint percentage (LP) and boll number per plant (BNPP), across 316 *G. hirsutum* accessions with diverse origins under four different salt conditions for two-years. Phenotypic variation of the three lint yield components under different salt environments showed that BNPP is a major limiting factor, causing the reduction of lint yield per plant (LYPP). Further, GWAS analysis was conducted by applying 57,413 SNPs to identify QTLs and candidate genes associated with LP, SBW and BNPP. In the current study, LP was systematically clarified as the most stable trait, and BNPP was most easily affected by salty field conditions. The results provide new insights into the mechanisms of salt stress, and how improvement of boll number with enhanced salt tolerance may improve cotton yield.

## Results

### Phenotypic variation of lint yield components under salt conditions

SBW, LP and BNPP were measured in 316 upland cotton accessions (Additional file 1: Table S1) grown under four different salt conditions (Additional file 2: Figure S1A and Additional file 3: Table S2). The average values of SBW, LP and BNPP traits ranged from 5.28 g to 6.84 g (Additional file 4: Table S3), 37.89 to 40.39% (Additional file 5: Table S4) and 5.01 to 7.74 (Additional file 6: Table S5) under the four different salt conditions, respectively. BNPP had the largest coefficients of variation (CV), ranging from 22.53 to 48.08%, and LP had the smallest (8.15%~ 12.68%). To decrease the environmental errors, we further evaluated the best linear unbiased prediction (BLUP) value of the three traits in the same salt conditions. The BLUP value showed that the distribution of SBW was 5~7 g (Additional file 2: Figure S1B) and LP at 35~45% (Additional file 2: Figure S1C). However, the BNPP showed a larger difference of 3~7 in salt conditions A and B, and 5~9 in salt conditions C and D (Additional file 2: Figure S1D).

**Fig. 1:**
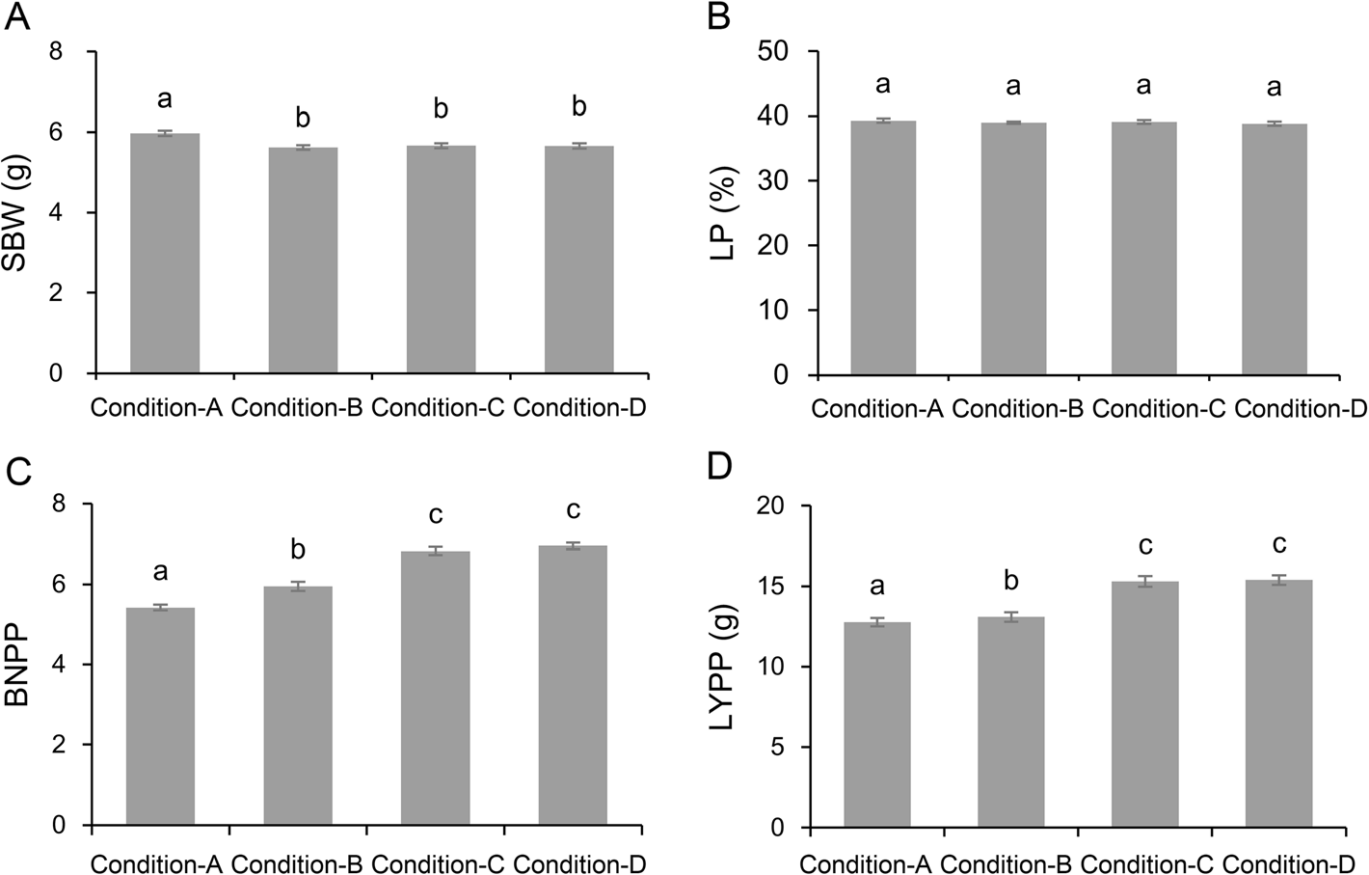
Comparison of different phenotypic data under four salt conditions. **a**. single boll weight (SBW). **b**. lint percentage (LP). **c**. boll number per plant (BNPP) **d**. lint yield per plant (LYPP). Statistical difference of single trait was calculated with paired-samples *t*-tests

Paired-samples *t-*tests were conducted to further investigate the phenotypic variation. The SBW was slightly higher in condition A than in conditions B, C and D (Fig. 1a); however, no significant difference in LP was detected between the four salt conditions (Fig. 1b). Most significantly, the BNPP decreased with the increase in salt concentration (Fig. 1c). In addition, the LYPP was also decreased with the increase in salt concentration, mainly due to changes in BNPP (Fig. 1d). We compared the differences in SBW, LP, BNPP and LYPP at the highest and lowest salt conditions, and found that SBW increased by 5.29%, LP remained unchanged, BNPP decreased by 17.75%, and LYPP decreased by 16.79%. Taken together, these results suggest that the lint yield of cotton is mainly affected by a decrease in boll number, although SBW was slightly increased. This might be due to severely low boll numbers under high salt conditions.

Correlation analysis was conducted on SBW, LP and BNPP under four salt conditions (Additional file 7: Figure S2). The results showed that there was little correlation among the three traits. However, LP values under different salt environments were highly correlated, with R values ranging from 0.75 to 0.83, and this was followed by SBW, which had R values ranging from 0.43 to 0.63. BNPP showed no or weak correlation in four salt conditions, further indicating that BNPP was largely affected by salt concentration.

### GWAS of lint yield related traits

Using the genotypic data of 57,413 high-quality SNPs [18], a GWAS was conducted for the three traits in different environments and the BLUP values, using six methods (“mrMLM”, “FASTmrMLM”, “FASTmrEMMA”, “pKWmEB”, “ISIS EM-BLASSO” and “pLARmEB”) of multi-loci mixed linear model (MLM) model in the R package “mrMLM” [19]. In total, 854 quantitative trait nucleotides (QTNs) on 26 chromosomes were identified as significantly associated with the three traits (Additional file 8: Table S6). We referenced the linkage disequilibrium (LD) in previous report [18] and calculated the average LD from each chromosome, further selected the lowest LD, about 200 kb, as the LD threshold, for merging QTNs into the same QTL. In total, 600 QTLs, including 151 of SBW, 417 of LP and 112 of BNPP, were detected 1446 times under different environments and BLUPs by six multi-loci MLM models (Table 1). For each trait, most QTLs (85 of SBW, 235 of LP and 65 of BNPP) were detected only once, implying that these QTLs are apt to be affected by environmental conditions (Additional file 9: Figure S3). To improve the reliability and stability of associated QTLs, we selected those that were detected three or more times across different methods or environments as stable QTLs for further analysis. As a result, 42, 91 and 25 QTLs were identified in SBW, LP and BNPP, respectively (Table 1).

**Table 1:** GWAS analysis of three traits under four salt conditions by multi-loci MLM model

Trait	Salt condition	Number of stable/total QTL	Detected count of stable/total QTL^a^
SBW	A	21/49	69/103
	B	12/39	56/90
	C	13/40	48/76
	D	18/47	50/87
	Total	42/151	223/356
LP	A	49/136	113/209
	B	56/188	131/276
	C	57/165	110/224
	D	50/106	97/159
	Total	91/417	451/868
BNPP	A	7/30	25/53
	B	9/31	32/60
	C	8/24	29/50
	D	8/34	27/59
	Total	25/112	113/222
Total		150/600	787/1446

^a^ indicated the detected counts of *QTLs* in different methods, years or replications. *SBW* single boll weight; *LP* lint percentage; *BNPP* boll number per plant. A, B, C, and D represented the total salt contents under the four soil environments, with 19 g/Kg (condition A), 10 g/Kg (condition B), 7 g/Kg (condition C), and 5 g/Kg (condition D), respectively

**Fig. 2:**
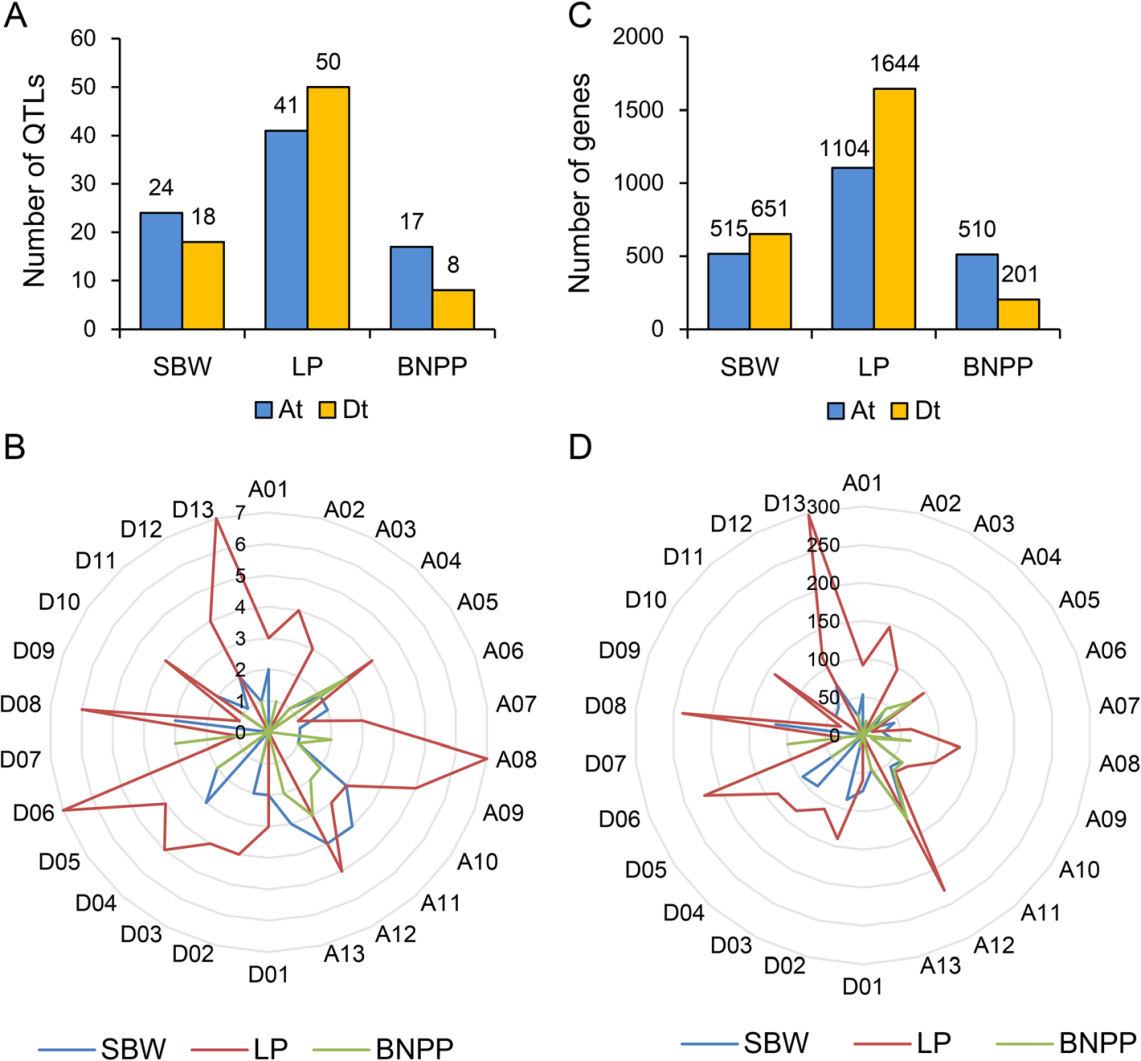
Genomic distribution of QTLs and candidate genes associated with the three traits. **a**. Numbers of QTLs on At and Dt sub-genome. **b**. Numbers of QTLs on 26 chromosomes. **c**. Numbers of candidate genes on At and Dt sub-genome. **d**. Numbers of candidate genes on 26 chromosomes

**Fig. 3:**
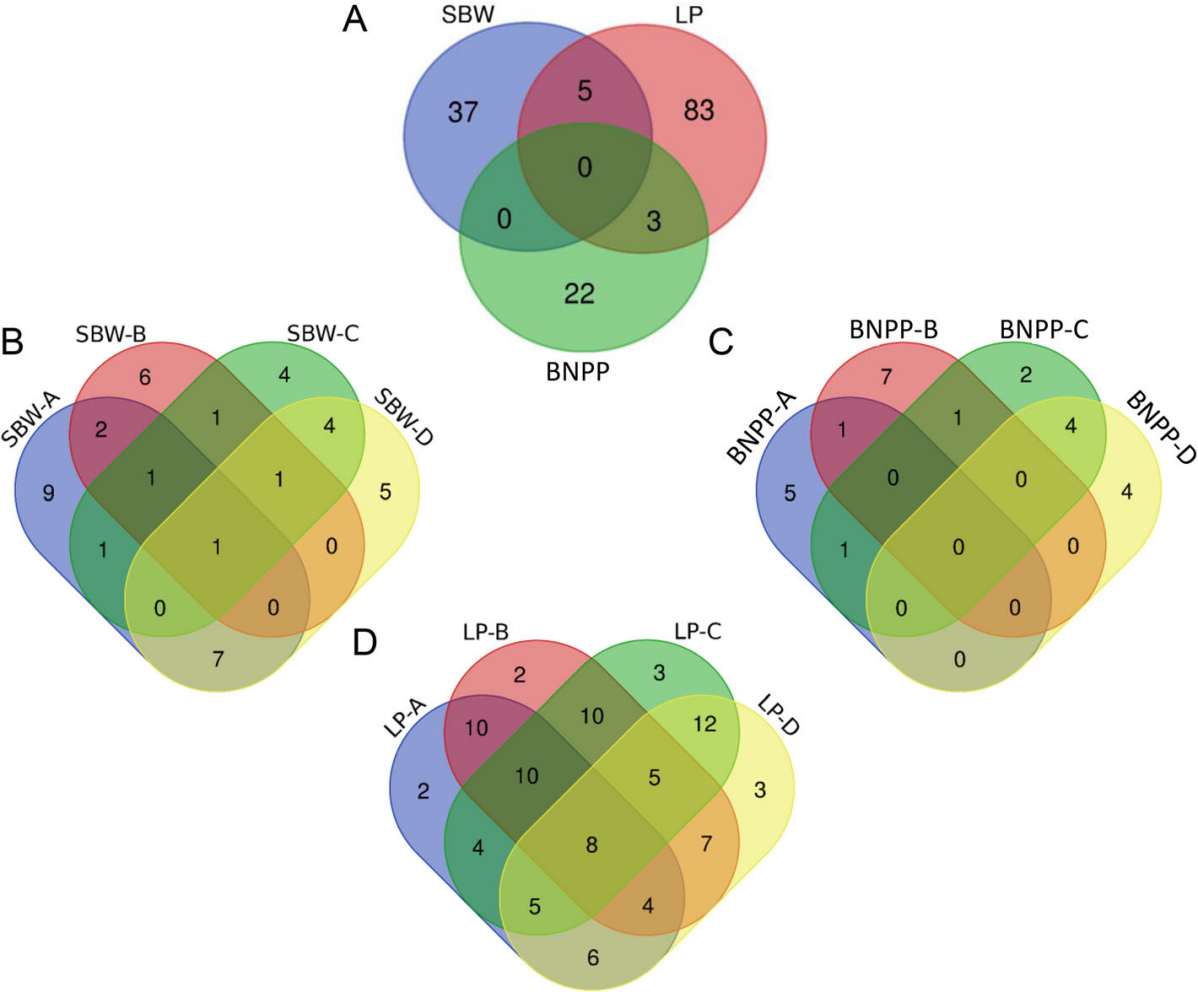
Venn diagram of QTLs associated with three traits under four different salt conditions. **a**. Venn diagram of QTLs among three traits. **b**. Venn diagram of QTLs associated with SBW in four salt conditions. **c**. Venn diagram of QTLs associated with LP in four salt conditions. **d**. Venn diagram of QTLs associated with BNPP in four salt conditions

The chromosomal distribution showed that these stable QTLs were widely distributed on 26 chromosomes, with more QTLs of SBW and BNPP located on the At sub-genome than on the Dt sub-genome, while QTLs of LP showed opposite (Fig. 2a). Most SBW QTLs were located on chromosomes A11 and A12, LP on A08, D06 and D13, and BNPP on A05, A12 and D07 (Fig. 2b). A Venn diagram of these stable QTLs showed that no co-localized QTL was detected within three traits and most QTLs were specific for individual traits (Fig. 3a), implying great differences in the genetic control of the three traits. We further analyzed the QTLs of each trait under different salt conditions. Only one QTL of SBW was detected under all four salt conditions, and no overlapping QTLs were detected in BNPP (Fig. 3b, c). However, most QTLs of LP were detected repeatedly under different salt conditions: there were 8 QTLs of LP detected simultaneously under all four salt conditions (Fig. 3d). These results suggest complex and variable regulatory mechanisms for SBW and BNPP under different salt conditions, but stable and highly heritable LP regulation against salt stress.

### Identification of candidate genes in QTLs

Potential candidate genes in these stable QTL regions were extracted based on the released *G. hirsutum* TM-1 genome [20]. In total, 1166, 2748, and 711 candidate genes were identified in the stable QTL regions for SBW, LP and BNPP, respectively (Fig. 2c), with most genes distributed on chromosomes A12, D13 and A12 for SBW, LP and BNPP, respectively (Fig. 2d). With GO analysis, the genes in QTL regions for SBW were found to be enriched in “embryo development” and “regulation of cell shape” (Additional file 10: Figure S4 and Additional file 11: Table S7). The genes from LP QTLs were enriched in several pathways, including hormone and ROS regulation pathways, such as “regulation of gibberellic acid mediated signaling pathway”, “positive regulation of reactive oxygen species metabolic process” and “brassinosteroid biosynthetic process”. They were also enriched in carbohydrate metabolism processes, such as “glucose metabolic process” and “hexose biosynthetic process”, which was consistent with the previous reports that these GO items play crucial roles in fiber development [11, 21]. In addition, we identified 14, 21 and 10 genes related to “Golgi vesicle transport”, “plant-type secondary cell wall biogenesis” and “glucose metabolic process”, respectively, however, none of these process-related genes were found in the QTL regions of BNPP or SBW (Additional file 12: Figure S5 and Additional file 11: Table S7). The function of genes associated with BNPP were mainly enriched in “mitotic cell cycle”, “ion transmembrane transport” and “polysaccharide catabolic process” (Additional file 13: Figure S6 and Additional file 11: Table S7), implying that BNPP is closely related to stress responses.

### Genes relevant to LP

Via tissue and organ transcriptome profiling [22], we identified 182 genes from LP QTLs with predominant expression during fiber development. Of them, 29, 35, 73 and 45 genes were highly expressed at 10 DPA, 15 DPA, 20 DPA and 25 DPA, respectively (Additional file 14: Figure S7 and Additional file 15: Table S8). Further, we focused on the genes in the regions of 8 stable QTLs under all four salt conditions and identified 10 genes predominantly expressed during fiber development (Additional file 16: Table S9). Of them, *Gh_A05G2488*, encoding an auxin transport facilitator family member called PIN-FORMED LIKES proteins (PILS), was located in a high frequency associated QTL (A05: 32377816–33,100,112) which was detected 21 times with multiple methods and environments. Auxin is essential for plant growth and development, including cotton fiber development. Prominent auxin carriers with fundamental importance during plant development are PIN-FORMED (PIN) proteins [23, 24]. Two genes, *Gh_D13G0342*, which encodes RAB GTPase homolog G3F (RABG3F), and *Gh_A10G2138*, which encodes prenylated RAB acceptor protein 1 (PRA1), were located on QTL (D13: 3297533–3,912,736) and QTL (A10: 99896949–100,396,471), respectively. Previous reports suggested that *RAB* genes play crucial roles in cell polarity growth, including elongation of pollen tubes [25], root hairs [26], and cotton fibers [27].

**Fig. 4:**
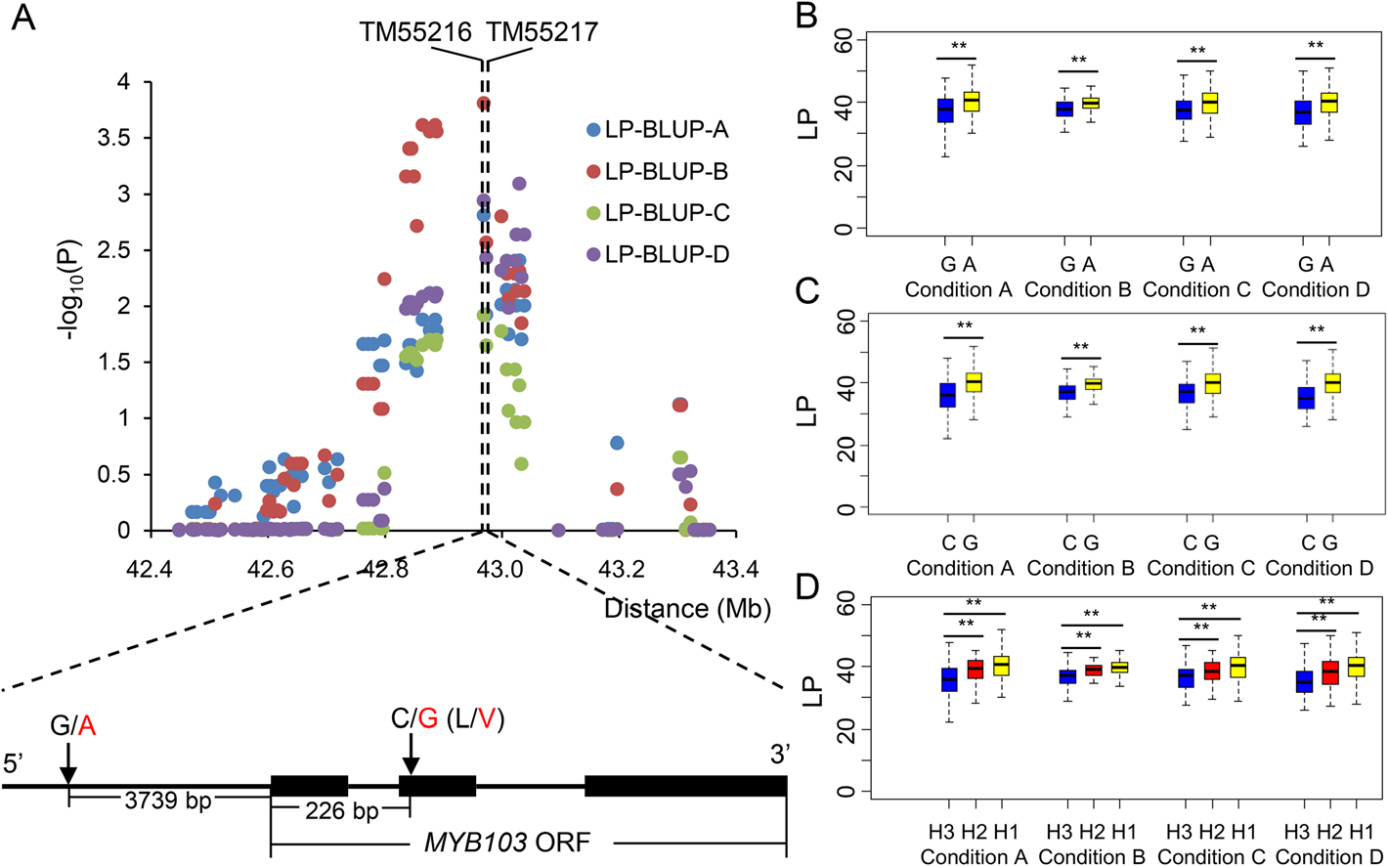
Haplotype analysis of the candidate gene *MYB103* on chromosome D03. **a**. Manhattan plots of SNPs around *MYB103* for the best linear unbiased prediction (BLUP) of LP across the four different salt conditions and the location of two QTNs related to *MYB103*. L indicated leucine and V indicated valine. **b**. Box plots for the phenotypic values of QTN TM55216. **c**. Box plots for the phenotypic values of QTN TM55217. **d**. Box plots for the phenotypic values of haplotype from the two QTN combinations. H1 indicated A-G genotype, H2 indicated A-C genotype, and H3 indicated G-C genotype. ** indicated *P* value at the 0.01 level with student’s *t* test, respectively

Cotton seed transfer cells are enriched in callose, which regulates fiber elongation and secondary wall thickening [28]. Zhang et al. (2007) showed that the transcription factor MYB103 affects callose dissolution during the anther development in *Arabidopsis* [29]. We found that *Gh_D03G1419*, which encodes a MYB103 transcription factor (named *GhMYB103*), was located in a high frequency associated QTL (D03: 42299450–43,529,568). Sequence analysis showed that two QTNs associated with LP were located in the 3739 bp upstream (TM55216, D03: 42969311) and exon (TM55217, D03: 42973276) regions of the gene, respectively (Fig. 4a). A single nucleotide mutation (from C to G) at the TM55217 locus led to a change of amino acid from leucine (L) to valine (V) (Fig. 4a). Through a Student’s *t* test, we found that the LP values with the A genotype in TM55216 were significantly higher than with the G genotype (Fig. 4b), and with the G genotype in TM55217 significantly higher than with the C genotype (Fig. 4c). The two QTNs could generate 3 haplotypes; H1: AG, H2: AC and H3: GC. The LP values with AG and AC haplotypes were significantly higher than that with GC haplotypes. However, there was no significant difference between AG and AC haplotypes, implying that QTN TM55216 might play more important roles in LP (Fig. 4d).

In addition, we integrated the LP QTLs with GWAS signals published in previous reports [11, 12], and found three QTLs (A12: 602614–743,324; D13: 58792627–59,289,811; A09: 4676815–5,076,815) that overlapped with GWAS signals. The three QTLs were detected in different salt conditions. Further, 10 genes from the QTL regions were identified to be predominantly expressed during fiber development (Additional file 16: Table S9). Of them, *ABP1* [30] and *CPK17* [31] were related to hypocotyl growth or fiber development.

Taken together, the characteristics of genes from these stable LP QTLs and their predominant expression during fiber development suggests that they could play an important role in the LP improvement in breeding practice.

### Genes relevant to BNPP

The QTLs of BNPP were easily affected by changes to the environment. No repeated QTLs were detected under all four salt conditions and most QTLs were identified in only one or two salt environments. For example, QTLs TM57617_TM57620 (D05: 24.3–24.8 Mb, detected with 16 times) and TM74225 (D10: 24.1–24.5 Mb, detected with 7 times) were identified in salt conditions C and D. However, QTL TM52041_TM52044 (D02: 49.2–49.7 Mb, detected with 6 times) was identified only in salt condition A, and QTL TM29006 (A08: 81.7–82.1 Mb, detected with 5 times) was identified only in salt condition B. These results indicate that the genes regulating the number of bolls varied in different salt conditions.

A large number of items related to ion transport were found to be enriched through GO analysis (Additional file 13: Figure S6 and Additional file 11: Table S7). In order to further explore the relationship between stress tolerance and boll number, we performed RNA-seq analysis using the salt stress transcriptome of *Gossypium* acc. TM-1. With the filter of FPKM ≥3, 500 genes (446 in roots and 395 in leaves) in QTL regions were obtained. With GO annotation and differential expression analysis, we focused on 40 salt-inducible stress response genes. Of them, 6 were commonly identified in roots and leaves (Additional file 17: Figure S8, Additional file 18: Table S10 and Additional file 19: Table S11). *Gh_A04G1216* encodes a high-affinity K^+^ transporter 1 (HKT1). *AtHKT1* limits root-to-shoot sodium transportation and is believed to be essential for salt tolerance in *Arabidopsis thaliana* [32]. *Gh_A05G3239* encodes a peroxidase superfamily protein (POD), which has been shown to play an important role in anti-oxidation under salt stress in cotton [33]. *Gh_A08G1183* encodes a mitogen-activated protein kinase (MAPK), which has been widely reported to be associated with salt tolerance in cotton [34]. In addition to the ion transport GO term, carbohydrate metabolism was active under salt stress, for example, 10 genes were identified in the enriched GO term “polysaccharide catabolic process” and 3 of them were differentially expressed under salt stress. Cell cycle regulation is of pivotal importance for plant growth and development [35]. A total of 33 genes were identified in the enriched GO term “cell cycle”, 8 of which were differentially expressed under salt stress. These candidate genes could contribute to increasing boll number under different salt environments in cotton.

**Fig. 5:**
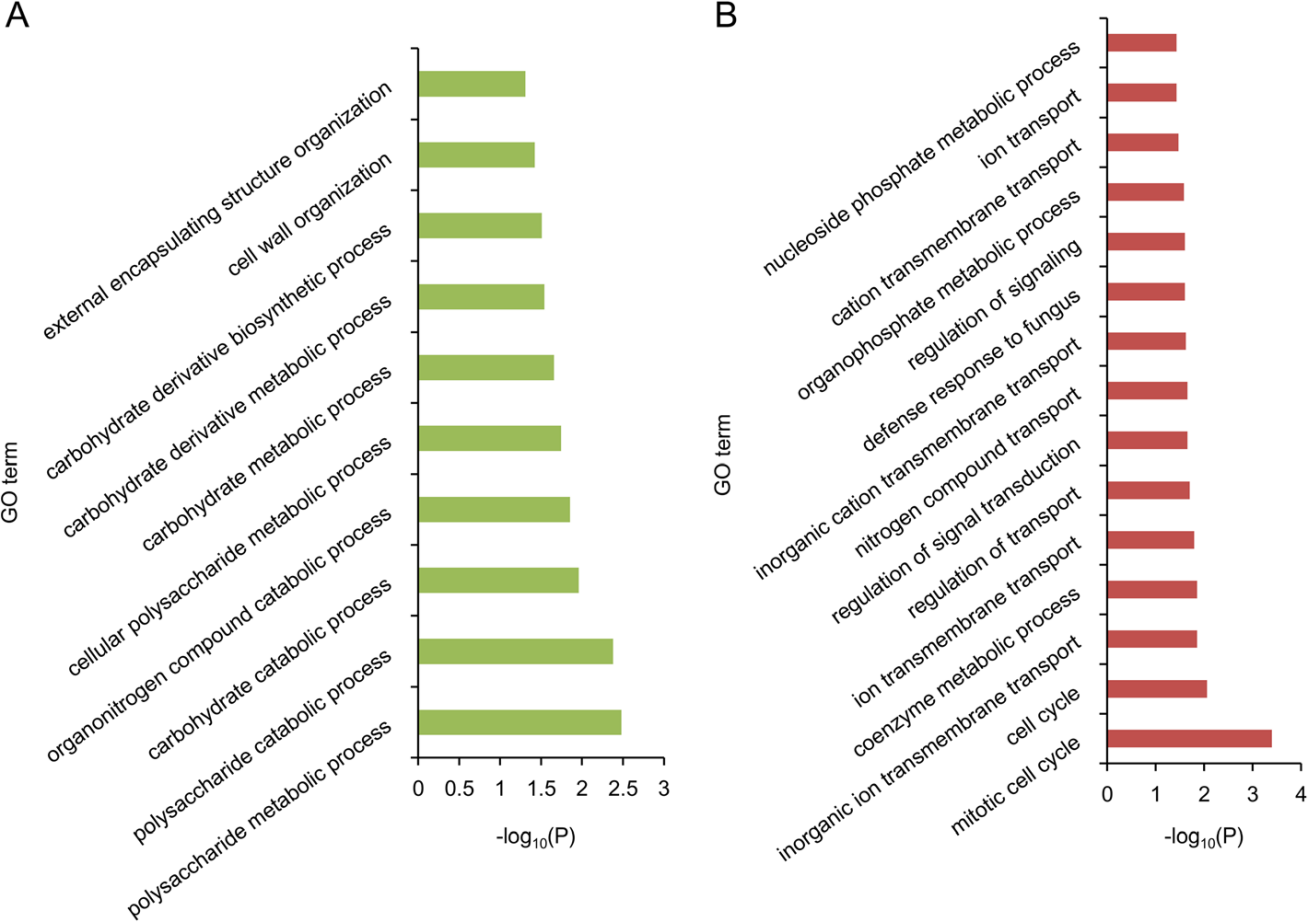
GO enrichment of genes associated with BNPP under salt condition A and D. **a**. salt condition A. **b**. salt condition D

In order to explore the key genes and mechanisms of boll number regulation under high and low salt conditions, we compared the genes located in the QTL regions of BNPP between conditions A and D. In total, 204 and 265 genes were identified under salt conditions A and D, respectively. GO enrichment analyses showed significantly different functional classification of genes between high and low salt conditions. GO terms related to “polysaccharide metabolic process”, “carbohydrate catabolic process” and “cell wall organization” were enriched under high salt condition A (Fig. 5a, Additional file 20: Table S12), while “cell cycle”, “ion transmembrane transport” and “regulation of signal transduction” were enriched under low salt condition D (Fig. 5b). This indicates that carbohydrate metabolism and cell structure maintenance play crucial roles under high salt conditions, while ion transport is a basal process that is more important under low salt conditions. Under high salt conditions, several candidate genes associated with BNPP were detected. *Gh_A11G1551* encodes a proline dehydrogenase 1 (ProDH1), also called early responsive to dehydration 5 (ERD5), which has been studied extensively, especially under abiotic stress [36]. For energy metabolism, *Gh_A05G1912* encodes an isoamylase 3 (ISA3), which contributes to starch breakdown. Under low salt conditions, three candidate genes were identified to play important roles in the balance between sodium and potassium. In detail, *Gh_A10G0441* encodes a potassium transporter 1 (KUP1) [37], *Gh_A12G0074* encodes a high affinity K^+^ transporter 5 (HAK5) [38] and *Gh_A12G0061* encodes a sodium hydrogen exchanger 2 (NHX2) [39].

## Discussion

With the decrease in arable land area and the deterioration of soil environments throughout the world, there is an urgent need to improve stress tolerance in crop plants. Xinjiang is the main cotton production area in China, but soil salinization levels in this area are high. Excavating elite alleles that can increase cotton yield under saline-alkali conditions is of great significance for breeding new varieties with high yield and stress resistance. With the development of high-throughput sequencing technology and new statistical methods, GWAS provides a fast and effective method for functional gene mining in plants. Several GWAS signals for vegetative growth index related to salt tolerance have been detected at the germination and seeding stages in previous studies [4, 17]. However, there are few GWAS studies on cotton yield traits under stress conditions. In the present study, we focused on three lint yield traits (SBW, LP and BNPP) and identified favorable associated QTLs and elite alleles under four salt conditions. The results provide new insights into the genetic basis of salt tolerance and the identification of novel alleles underlying the variation in salt-tolerance traits and candidate genes, allowing the acceleration of the progress of cotton tolerance breeding.

Cotton yield decreases under salt stress. Previous studies have reported that SBW, LP and BNPP are remarkably decreased under salt stress conditions [40–42]. In the present study, we selected four different field soil salt environments with two year replicates to investigate the lint yield components of cotton accessions. Through phenotypic analysis, LP did not change significantly under different salt conditions. SBW was slightly higher in the highest salt concentration than in other three lower salt concentrations, and this might be relevant to the decreased BNPP. It is worth noting that BNPP decreased significantly with the increase in total salt content, which results in the reduction of cotton lint yield. Overall, BNPP is the most susceptible factor to salt stress compared to the other two traits investigated. Compared with the lowest salt condition, the highest salt condition could cause 16.79% loss of LYPP, which was mainly caused by the reduction in BNPP. Hence, it is important to maintain the boll number in cotton under salt stress to maintain high cotton lint yield. In addition, there was less correlation among SBW, LP and BNPP traits, implying that different biological processes regulate the three traits under salt stress.

Increasing yield is a major goal of cotton breeding programs. LP is an important yield component and a critical economic index for cotton cultivars. Although phenotypic analysis showed no significant change in LP under different salt conditions in present study, GWAS results indicated that there were fewer overlapped QTLs compared with the previous reports in non-salt conditions [11, 12], implying that some specific genes contribute to the improvement of LP under salty conditions. We also found a large number of QTLs associated with LP on chromosomes A08 and D08, which were reported to contain many QTLs or key genes related to fiber development [11, 12]. However, the distribution of QTLs associated with LP was quite different from that reported by Su et al. (2016) [43]. Taken together, these results suggest that LP is a complex quantitative trait, and the majority of loci detected in our study were novel and might be related to salt stress. In particular, 8 QTLs were identified simultaneously in four salt conditions, which could contribute to the increase in LP under salt stress. Further, we identified 10 genes that were closely related to fiber development in these stable LP QTLs, such as *PILS*, *RAB* and *MYB*. In *Arabidopsis*, overexpressing *PILS* genes reduced hypocotyl and root growth [44]. However, auxin accumulation can promote cell initiation (− 2 to 2 DPA) in the fiber cell. Transgenic assay showed that ovule-specific suppression of multiple *GhPIN* genes inhibited both fiber initiation and elongation in cotton [45], indicating that *Gh_A05G2488* plays an important role in fiber development. The RAB and RAB acceptor protein also have potentially important functions in cotton fiber development. In *Arabidopsis*, *RabA4d* is necessary for the proper regulation of pollen tube growth. Loss of *RabA4d* leads to the destruction of pollen tube growth and changes in the structure of the cell wall [46]. In addition, PRA1 domain proteins are small transmembrane proteins that regulate vesicle trafficking as receptors of Rab GTPases. AtPRA1 proteins were localized to the endoplasmic reticulum, Golgi apparatus, and endosomes/prevacuolar compartments, indicating a function in both secretory and endocytic intracellular trafficking pathways [47]. MYB transcription factor is also well known to play crucial roles in fiber development. *GhMYB212* RNAi plants (GhMYB212i) accumulated less sucrose and glucose in developing fibers, and had shorter fibers and a lower lint index [48]. Expression analysis of the *MYB* family showed that *GhMYB103* was highly expressed in 25 DPA seed fibers and was correlated with cellulose synthesis [49]. In our study, two significant QTNs located in the upstream and exon regions of *GhMYB103*, respectively, were found to cause gene expression level changes and amino acid variation, and then affect the LP of cotton. In addition, the SNP located in the upstream regulatory region of *GhMYB103* might play a more important role in LP following haplotype analysis. Although many genes related to LP have been identified by GWAS analysis, candidate genes in this study may play a more important role in improving LP under salt stress. We also identified three QTLs that were co-localized with the LP loci reported previously from GWAS analysis, and further identified 10 candidate genes in the QTL regions. These studies could provide genetic resources for improving LP in both salt and normal environments.

GWAS analyses of BNPP are relatively rare, especially under salt stress. Our studies showed that salt stress can lead to a significant decrease in boll number per plant, which was consistent with previous reports [40, 50], indicating that boll number is the first limiting factor for increasing cotton lint yield under stress environments. We also found that the number of repeated QTLs associated with BNPP was low under different salt conditions, implying a complex regulatory mechanism for BNPP. GO analysis showed that genes associated with BNPP are mainly involved in “mitotic cell cycle”, “ion transmembrane transport” and “polysaccharide catabolic process”. Of them, a large number of ion transport related processes are enriched, which suggests that excellent ion transport capacity plays a key role in the salt tolerance of cotton. Na^+^ accumulation can lead to ion poisoning, which induces the decline of biomass and yield losses in crop plants [1]. Maintaining ion homeostasis by ion uptake and compartmentalization is crucial for plant growth during salt stress. With RNA-seq analysis, we found that *HKT1*, which is known to play a role in the removal of Na^+^ from the xylem and bring it back to the root, was down-regulated under salt stress conditions. Overexpression of *HKT1* in roots can decrease Na^+^ accumulation in the shoot and significantly improve salt tolerance in *Arabidopsis thaliana* [51]. Interestingly, *HKT1* was also found to be down-regulated under salt stress conditions in *G. davidsonii*, a cotton D-genome diploid species with important properties of salinity stress resistance [52]. This suggests that the function of *HKT1* could be improved in cotton to increase stress tolerance. We also found no overlapped QTLs associated with BNPP by comparing the QTLs under high and low salt conditions, implying a complex regulatory mechanism under different salt conditions. The enriched genes under high salt conditions were mainly related to energy metabolism and maintenance of cell morphology. High salt stress can lead to a decrease in photosynthetic efficiency of plants [53]. Under non-stressed conditions, plants use the majority of the energy to maintain vegetative and reproductive growth. However, plants need to allocate more energy to resist stress with increasing salt concentrations [1]. In addition, high salt concentration can also increase osmotic stress, and plants need to synthesize more osmolytes to maintain cell morphology. In this study, we found that *ISA3* played crucial roles in energy metabolism and *ProDH1* contributed to maintenance of cell morphology. *ISA3* acts at the surface of the starch granule and removes short branches from the granule surface that can improve the rate of the starch breakdown. *Atisa3* mutants have more leaf starch and a slower rate of starch breakdown than wild-type plants [54]. To counteract osmotic stress caused by salt stress, some plants accumulate several kinds of compatible osmolytes, such as proline, glycine betaine, and sugar alcohols, to protect macromolecules and maintain the osmotic equilibrium inside and outside cell membrane [55]. The expression of *ProDH2*, a highly homologous gene of *ProDH1*, can promote proline accumulation under stress conditions [56]. In addition, the enriched genes under low salt conditions were mainly related to ion transport. As a salt-tolerant crop, cotton suffers less salt damage under low salt conditions than other plants, which may be due to efficient ion transport capacity. In this study, *KUP1*, *HAK5* and *NHX2* were identified to contribute to ion homeostasis. This suggests that an active sodium and potassium ion exchange capacity at low salt concentrations is the basis of salt tolerance in cotton. In addition, these results also reflect the different demands for stress resistance under different salt stress conditions in cotton.

Several reports have suggested that lint yield can be improved by altering the expression of salt-tolerance genes in cotton. For example, overexpressing *AvDH1* in cotton can decrease membrane ion leakage, and increase the activity of superoxide dismutase, leading to salinity tolerance and increased yield [50]. Overexpression of *SNAC1*, which belongs to the stress-related NAC superfamily of transcription factors, could improve drought and salt tolerance by enhancing root development and reducing transpiration rate [57]. In this study, we first report phenotypic and GWAS analysis of three lint yield components in cotton under salt stress. We found that BNPP was the most important factor for cotton lint yield in saline-alkali soil environments. Further, we identified a large number of elite alleles that contributed to the improvement of lint yield under salt conditions. These findings will help us understand the mechanisms of salt tolerance in cotton and provide improvements for breeding cultivars in saline-alkali soil environments.

## Conclusions

Elucidating the genetic variation of cotton lint yield components in saline-alkali soils has more practical value for saline-alkali soil utilization. In our study, phenotypic analysis of 316 upland cotton accessions under different salt conditions suggests that the lint yield of cotton is mainly affected by a decrease in boll number. Through GWAS analysis, we identified 42, 91 and 25 stable QTLs for SBW, LP and BNPP, respectively. Further, 10 candidate genes including *PILS*, *RAB* and *MYB*, being closely related to fiber development for LP improvement, and 40 candidate genes, such *HKT1*, *POD* and *MAPK*, with great significance for the increase of boll number in salt environment, were identified, respectively. In addition, different regulatory mechanisms for BNPP at high and low salt conditions were enriched, implying a complex regulation under different salt conditions. This study provides new insights for understanding the mechanisms of cotton salt stress, and contributes gene resources for increasing cotton yield by improvement of boll number under salt stress.

## Methods

### Plant materials and field experiments

A total of 316 upland cotton accessions, comprising 303 cultivars/lines collected from different regions of China and 13 landraces introduced from the United States, were used in this study (Additional file 1: Table S1). All accessions were collected and preserved by Nanjing Agricultural University, China. All necessary permits for planting and investigating the set of natural population were obtained from Nanjing Agricultural University, China.

In 2016 and 2017, the 316 upland cotton accessions were planted in four different salt field concentrations in Xinjiang Agricultural University Experimental Station (43°20′~ 45°20′E, 84°45′~ 86°40′N). All necessary permits for the field evaluations of these accessions were obtained from Xinjiang Agricultural University, China. The soil total salt contents were measured with five point sampling for each environment. In detail, the total salt contents in the four environments were 19 g/Kg (condition A), 10 g/Kg (condition B), 7 g/Kg (condition C), and 5 g/Kg (condition D), with two replicate plots for each salt condition (Additional file 2: Figure S1A and Additional file 3: Table S2). With a wide/narrow row alternation plantation mode (10 cm for narrow row and 66 cm for wide row), each accession was grown in two rows with a 2 m row length and 0.10 m between plants for each plot. Drip fertilization beneath mulched film was used for plant growth. Other agronomic practices were same for all treatments.

### Phenotype investigation and data analysis

Ten plants for each accession in each plot were selected randomly from the middle of each row and tagged for identification to allow the recording of data for SBW, LP and BNPP. At plant maturity (approximately 70% boll open), BNPP was counted with ten biological replicates. A total of 20 well developed open boll samples (2 bolls per plant) from the middle branches of tagged plants were harvested and weighed for SBW and calculation of LP. To reduce environmental influences, the best linear unbiased predictors (BLUPs) based on a mixed linear model for the three traits under each salt condition were estimated using the function of ‘lmer’ in the lme4 package [58]. In order to explore the effect of salt concentration on cotton lint yield, LYPP was calculated by multiplying SBW, LP and BNPP.

Paired-samples *t*-tests and correlation analysis among different salt conditions and traits were performed using SPSS software. Visualization of correlation analysis was performed using the R package “Performance Analytics”.

### GWAS analysis

Genomic DNA of the 316 cotton accessions was extracted according to the method described by Paterson et al. (1993) [59]. A CottonSNP80K array was used to genotype the 316 cotton accessions. The SNP genotyping and population structure were reported in our previous study [18]. A total of 57,413 SNPs (calling rate ≥ 0.9 and minor allele frequency (MAF) ≥ 0.05) were used for GWAS analysis. To explore the SNP-trait association, a multi-locus random-SNP-effect mixed linear model (mrMLM) [19] was employed using the R package “mrMLM” with the following parameters: Critical *P*-value in rMLM: 0.001; Search radius of candidate gene (Kb): 100; and Critical LOD score in mrMLM: 3. The Q + K model was used. A population structure (Q) matrix was calculated using admixture 1.3 with k = 3, and a kinship (K) matrix was calculated using the R package “mrMLM”. The BLUP values and single environments of three traits under different salt conditions were individually used for the GWAS.

### QTLs and candidate gene identification

We referenced the linkage disequilibrium (LD) published in a previous report [18] and calculated the average LD from each chromosome, and selected the lowest LD, about 200 kb, as the LD threshold, for merging QTNs into the same QTL. If the distance between two QTNs was less than 200 kb, these were merged into a single QTL. The QTLs of each trait discovered three or more times using different methods or in different environments were selected as stable QTLs. Putative candidate genes located in the stable QTL regions were extracted by self-written shell scripts from the reference genome TM-1 [20]. Gene ontology (GO) analysis was implemented using AgriGO V2.0 with the SEA method [60].

### RNA-seq analysis

To determine which genes are related to lint yield, the transcriptome profiles of TM-1 tissues were download from NCBI Sequence Read Archive collection PRJNA490626 [22]. Expression patterns in twenty three tissues or development stages, comprising root, stem, leaf, petal, torus, sepal, bract, anther, filament, pistil, ovule and fiber tissues at − 3, 0, 1, 3, 5 days post anthesis (DPA), ovules at 10, 15, 20, and 25 DPA, and fiber tissues at 10, 15, 20, and 25 DPA, were identified by calculating Z-scores. To investigate the genes related to stress tolerance, the transcriptome profiles of TM-1 roots and leaves treated by salt and control were downloaded from NCBI Sequence Read Archive collections PRJNA532694 and PRJNA490626, respectively. Both of the RNA-seq reads were mapped to the *G. hirsutum* acc. TM-1 genome using a Tophat spliced aligner with default parameters [61]. The genome-matched reads from each library were assembled with Cufflinks [62]. Cuffmerge was then used to merge the individual transcript assemblies into a single transcript set. Lastly, Cuffdiff was used to detect differentially expressed genes (DEGs) with a cutoff of 0.05 q-value. Three biological replicates from each sample were used for RNA-seq experiments. The heatmap of predominantly expressed genes and salt stress response genes were produced with Mev software (http://mev.tm4.org).

## Supplementary information

**Additional file 1 Table S1.** Information on 316 cotton accessions used in this study. (XLSX 20 kb)

**Additional file 2 Figure S1.** Sketch map of soil salt concentration and distribution of the phenotypic data for three lint yield components. A: Sketch map of soil salt distribution with different total salt content. The experimental field was divided into eight parts including four different total salt content with two replications for each salt condition. B-D: Distribution of phenotypic data of single boll weight (B), lint percentage (C) and boll number per plant (D) under four salt conditions. (TIFF 469 kb)

**Additional file 3 Table S2.** Measurement of total soil salt content in four different environments. (XLSX 11 kb)

**Additional file 4 Table S3.** Phenotypic data statistics of single boll weight under four salt conditions. (XLSX 10 kb)

**Additional file 5 Table S4.** Phenotypic data statistics of lint percentage under four salt conditions. (XLSX 10 kb)

**Additional file 6 Table S5.** Phenotypic data statistics of boll number per plant under four salt conditions. (XLSX 10 kb)

**Additional file 7 Figure S2.** Correlation analysis among three lint yield components and under different salt conditions for each trait. The red boxes indicated the correlation among three lint yield components. The green boxes indicated the correlation among different salt conditions for each trait. The number in these boxes indicated correlation coefficient (R valve). *, **, and *** indicated *P* value at the 0.05, 0.01 and 0.001 levels, respectively. (TIFF 2605 kb)

**Additional file 8 Table S6.** QTNs and QTLs of single boll weight, lint percentage and boll number per plant detected by multi-loci MLM model. (XLSX 151 kb)

**Additional file 9 Figure S3.** Distribution on detected times for 600 associated QTLs from three lint yield components, respectively. The x-axis represents the detected times; y-axis represents the number of QTLs. (TIFF 216 kb)

**Additional file 10 Figure S4.** The enriched biological processes of candidate genes associated with single boll weight. (TIFF 230 kb)

**Additional file 11 Table S7.** GO enrichment of genes associated with the three traits. (XLSX 21 kb)

**Additional file 12 Figure S5.** The enriched biological processes of candidate genes associated with lint percentage. (TIFF 552 kb)

**Additional file 13 Figure S6.** The enriched biological processes of candidate genes associated with boll number per plant. (TIFF 430 kb)

**Additional file 14 Figure S7.** Heatmap of predominant expressed genes associated with lint percentage in cotton fiber development. The number indicated different tissues or development stages, 1: root; 2: stem; 3: leaf; 4: petal; 5: torus; 6: sepal; 7: bract; 8: anther; 9: filament; 10: pistil; 11: -3DPA ovule and fiber; 12: 0DPA ovule and fiber; 13: 1DPA ovule and fiber; 14: 3DPA ovule and fiber; 15: 5DPA ovule and fiber; 16: 10DPA ovule; 17: 15DPA ovule; 18: 20DPA ovule; 19: 25DPA ovule; 20: 10DPA fiber; 21: 15DPA fiber; 22: 20DPA fiber; 23: 25DPA fiber. (TIFF 586 kb)

**Additional file 15 Table S8.** Expression pattern of 182 predominant expressed genes during fiber development in different tissues and organs. (XLSX 64 kb)

**Additional file 16 Table S9.** Candidate genes related to lint percentage identified in stable QTLs. (XLSX 11 kb)

**Additional file 17 Figure S8.** Heatmap of candidate genes related to boll number per plant under salt stress. The stress response genes located in QTLs were salt-inducible in roots (A) and leaves (B) under salt stress. The gene names marked in red represent differential expression in both roots and leaves. (TIFF 639 kb)

**Additional file 18 Table S10.** Expression pattern of 19 candidate genes related to boll number per plant under salt stress in roots. (XLSX 13 kb)

**Additional file 19 Table S11.** Expression pattern of 27 candidate genes related to boll number per plant under salt stress in leaves. (XLSX 13 kb)

**Additional file 20 Table S12.** GO enrichment of genes associated with boll number per plant under salt condition A and D. (XLSX 12 kb)

## Data Availability

RNA-Seq data in this study have been deposited at the National Center of Biotechnology Information (NCBI, http://www.ncbi.nlm.nih.gov/) under the accessions PRJNA490626, PRJNA532694 and PRJNA490626.

## Abbreviations

BLUP: The best linear unbiased prediction
BLUPs: The best linear unbiased predictors
BNPP: Boll number per plant
CV: Coefficients of variation
GBS: Genotyping by sequencing
GO: Gene ontology
GWAS: Genome-wide association studies
LD: Linkage disequilibrium
LP: Lint percentage
LYPP: Lint yield per plant
MAF: Minor allele frequency
QTLs: Quantitative trait loci
QTNs: Quantitative trait nucleotides
SBW: Single boll weight
SNP: Single nucleotide polymorphism
SSR: Simple sequence repeat
