# Supplementary figures and images for: Genome-wide association reveals genetic variation of lint yield components under salty field conditions in cotton (Gossypium hirsutum L.)
Source: BMC Plant Biol. 2020 Jan 14;20:23. doi: 10.1186/s12870-019-2187-y (PMC6961271; doi:10.1186/s12870-019-2187-y)

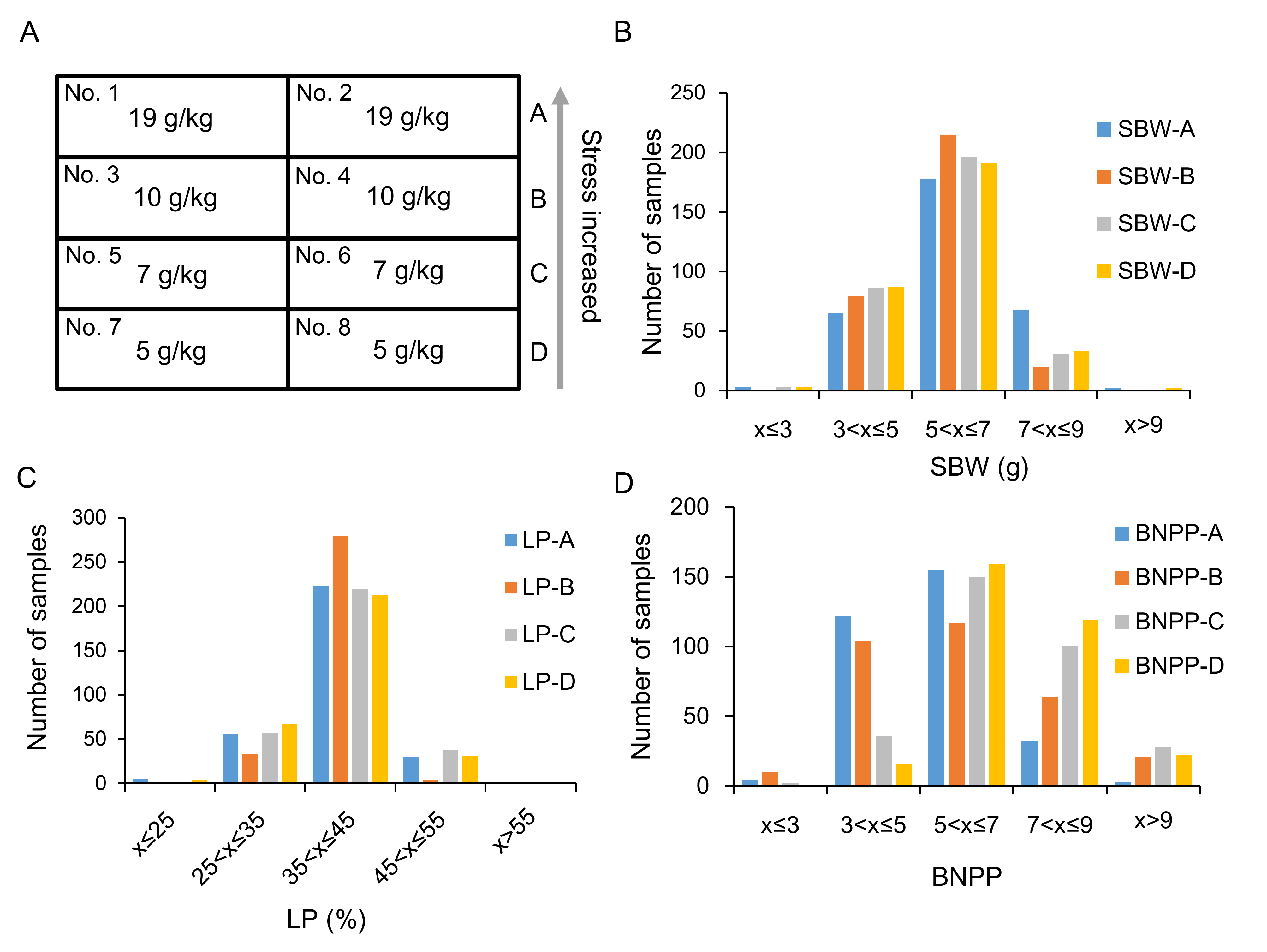

Supplement: Supplementary file 2 — Additional file 2 Figure S1. Sketch map of soil salt concentration and distribution of the phenotypic data for three lint yield components. A: Sketch map of soil salt distribution with different total salt content. The experimental field was divided into eight parts including four different total salt content with two replications for each salt condition. B-D: Distribution of phenotypic data of single boll weight (B), lint percentage (C) and boll number per plant (D) under four salt conditions. (TIFF 469 kb) [file 12870_2019_2187_MOESM2_ESM.tif]

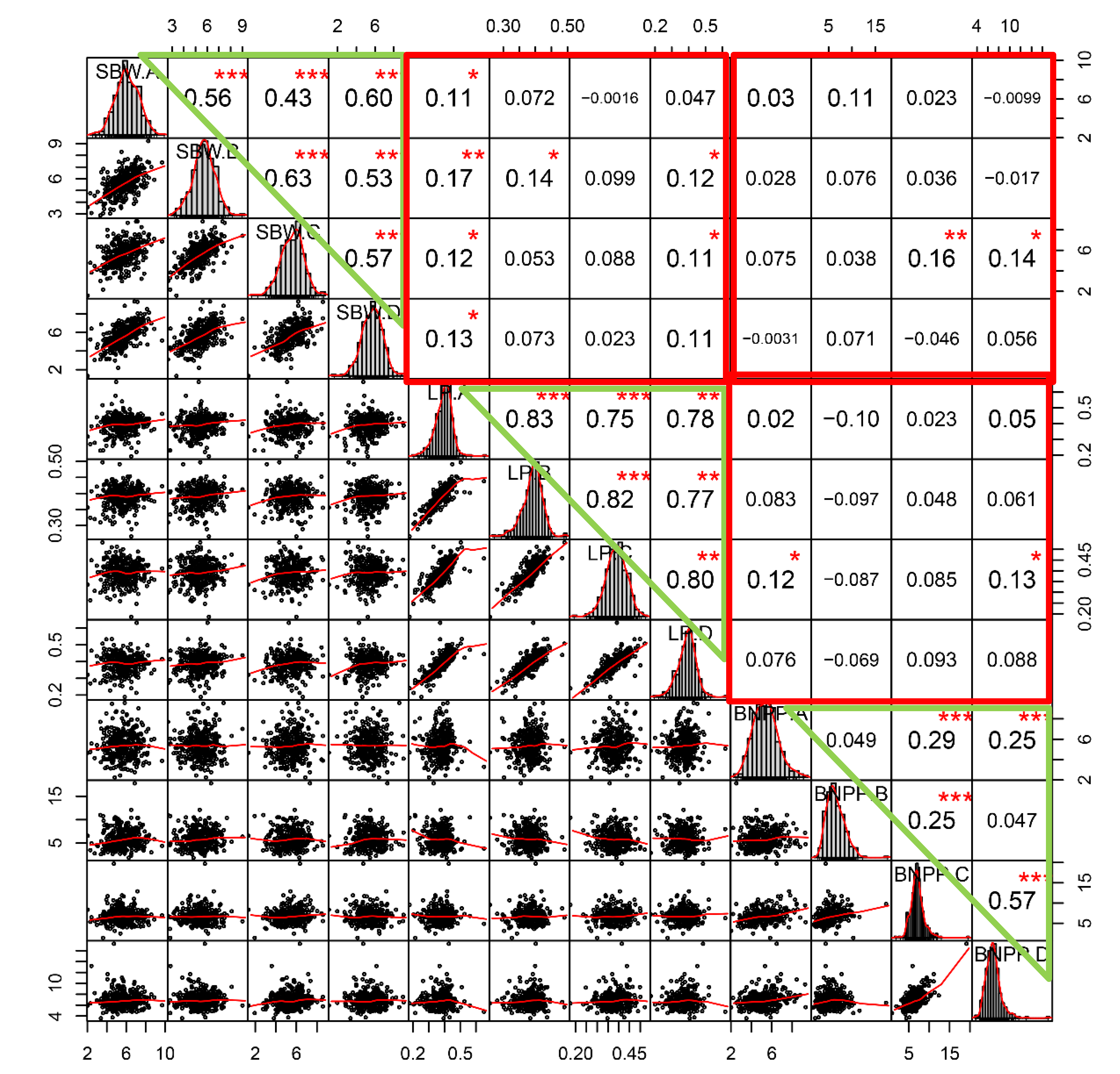

Supplement: Supplementary file 7 — Additional file 7 Figure S2. Correlation analysis among three lint yield components and under different salt conditions for each trait. The red boxes indicated the correlation among three lint yield components. The green boxes indicated the correlation among different salt conditions for each trait. The number in these boxes indicated correlation coefficient (R valve). *, **, and *** indicated P value at the 0.05, 0.01 and 0.001 levels, respectively. (TIFF 2605 kb) [file 12870_2019_2187_MOESM7_ESM.tif]

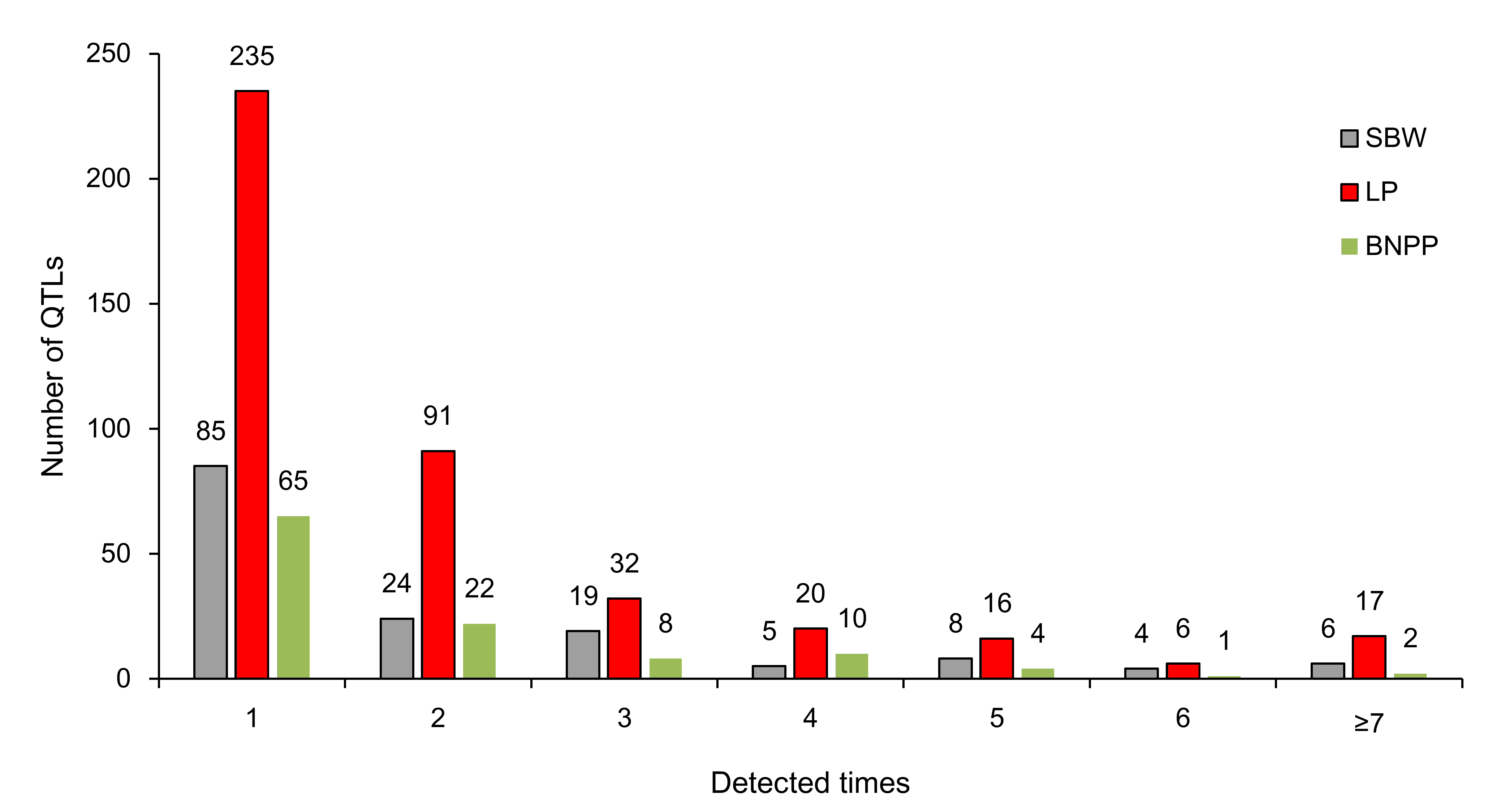

Supplement: Supplementary file 9 — Additional file 9 Figure S3. Distribution on detected times for 600 associated QTLs from three lint yield components, respectively. The x-axis represents the detected times; y-axis represents the number of QTLs. (TIFF 216 kb) [file 12870_2019_2187_MOESM9_ESM.tif]

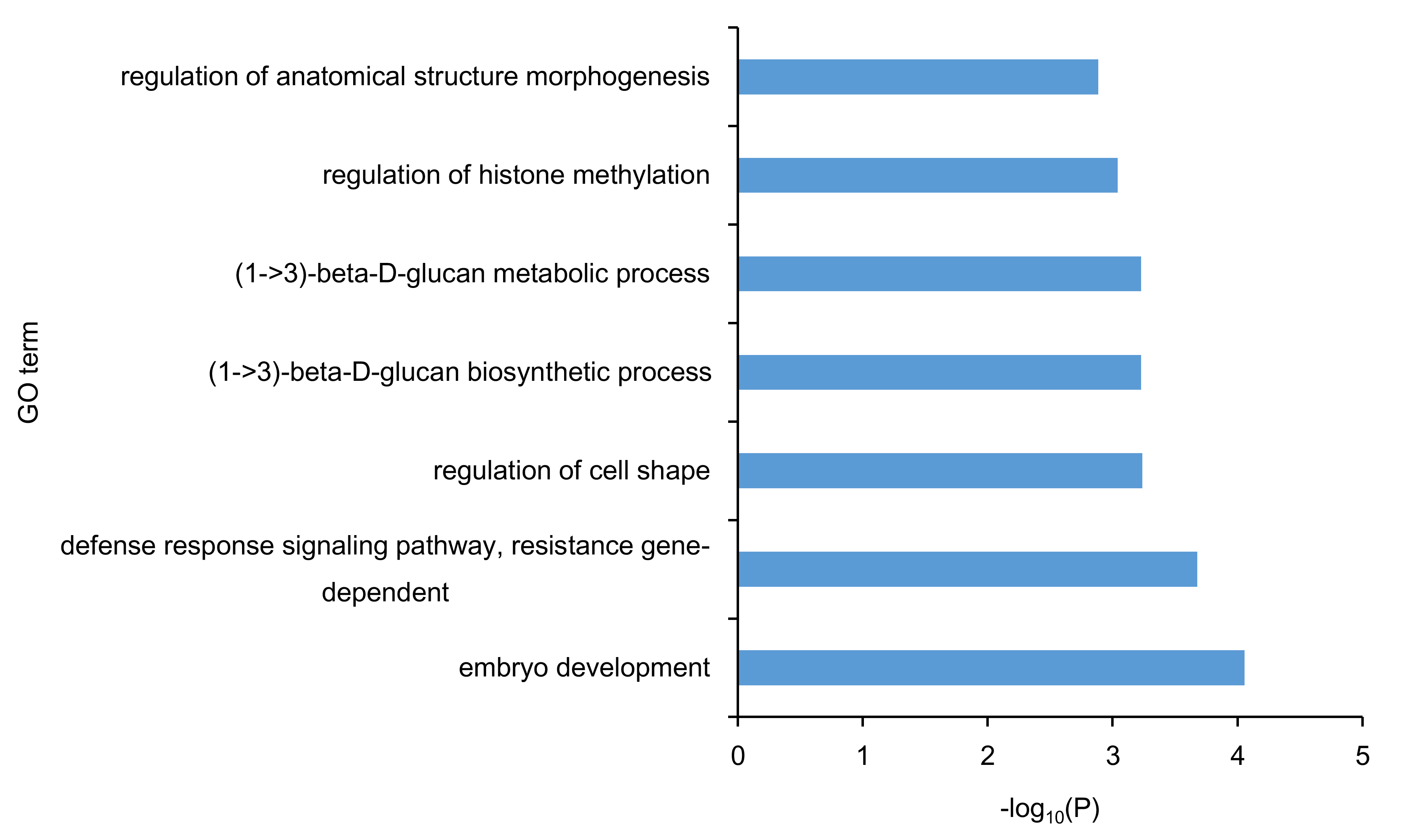

Supplement: Supplementary file 10 — Additional file 10 Figure S4. The enriched biological processes of candidate genes associated with single boll weight. (TIFF 230 kb) [file 12870_2019_2187_MOESM10_ESM.tif]

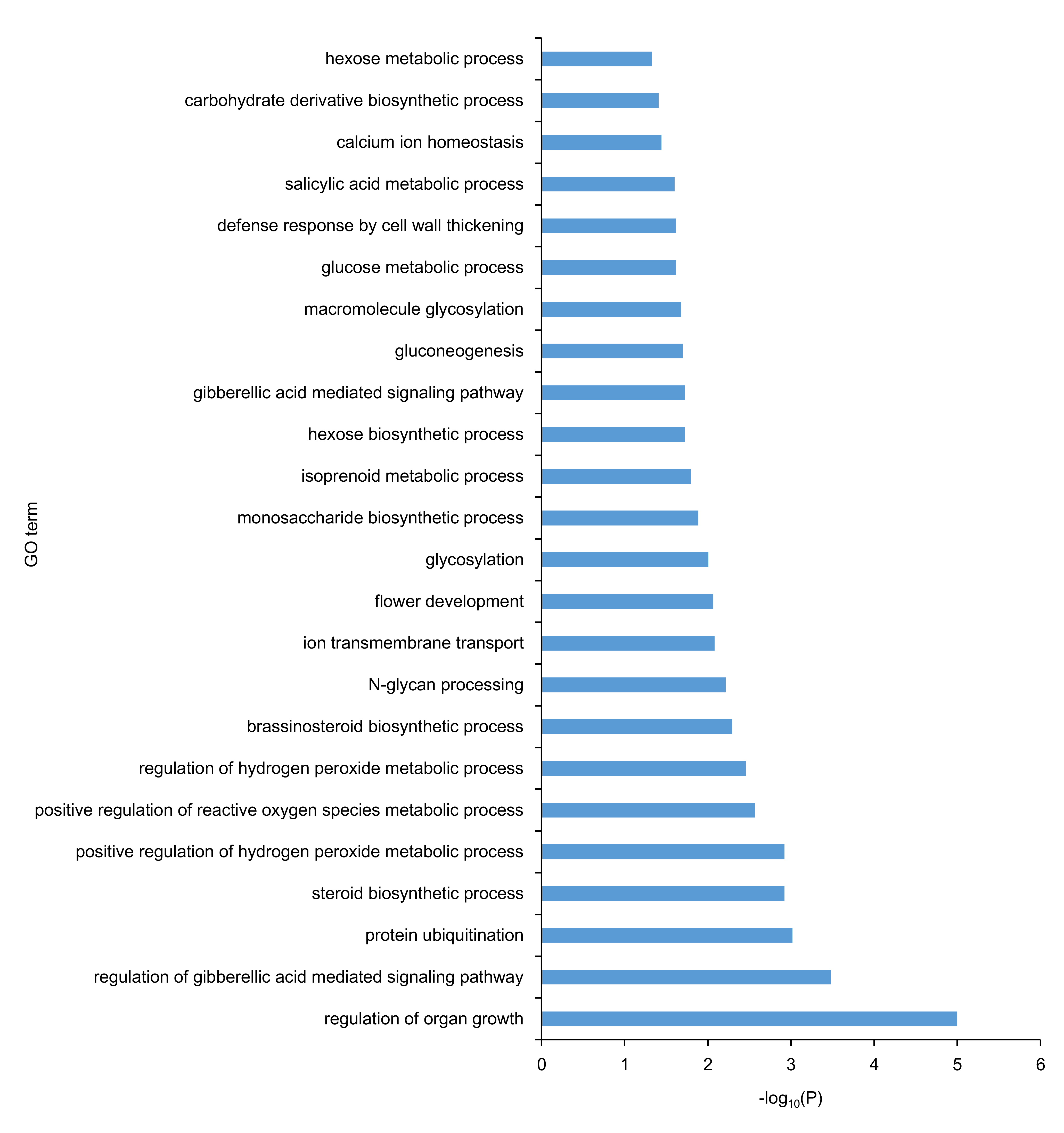

Supplement: Supplementary file 12 — Additional file 12 Figure S5. The enriched biological processes of candidate genes associated with lint percentage. (TIFF 552 kb) [file 12870_2019_2187_MOESM12_ESM.tif]

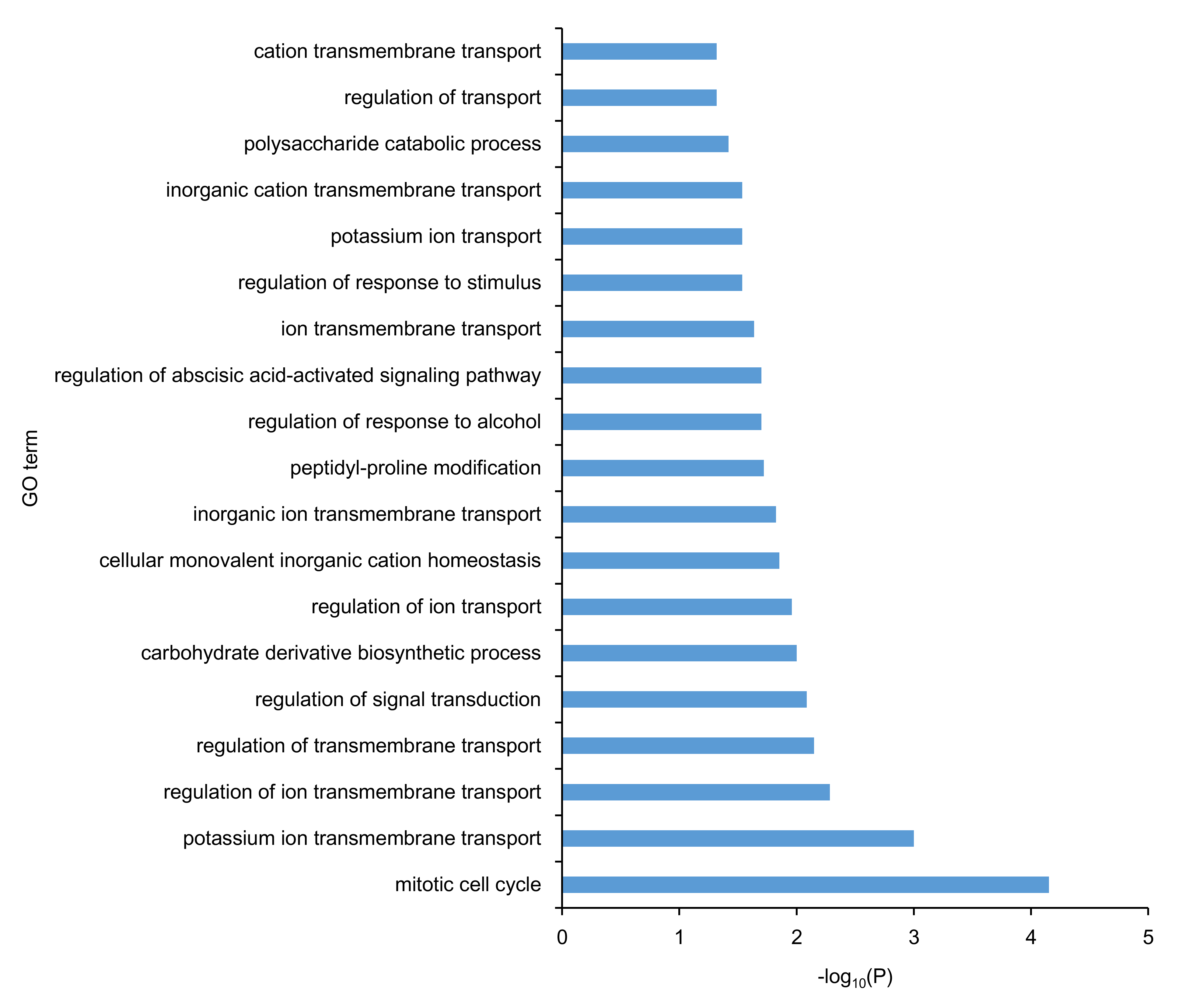

Supplement: Supplementary file 13 — Additional file 13 Figure S6. The enriched biological processes of candidate genes associated with boll number per plant. (TIFF 430 kb) [file 12870_2019_2187_MOESM13_ESM.tif]

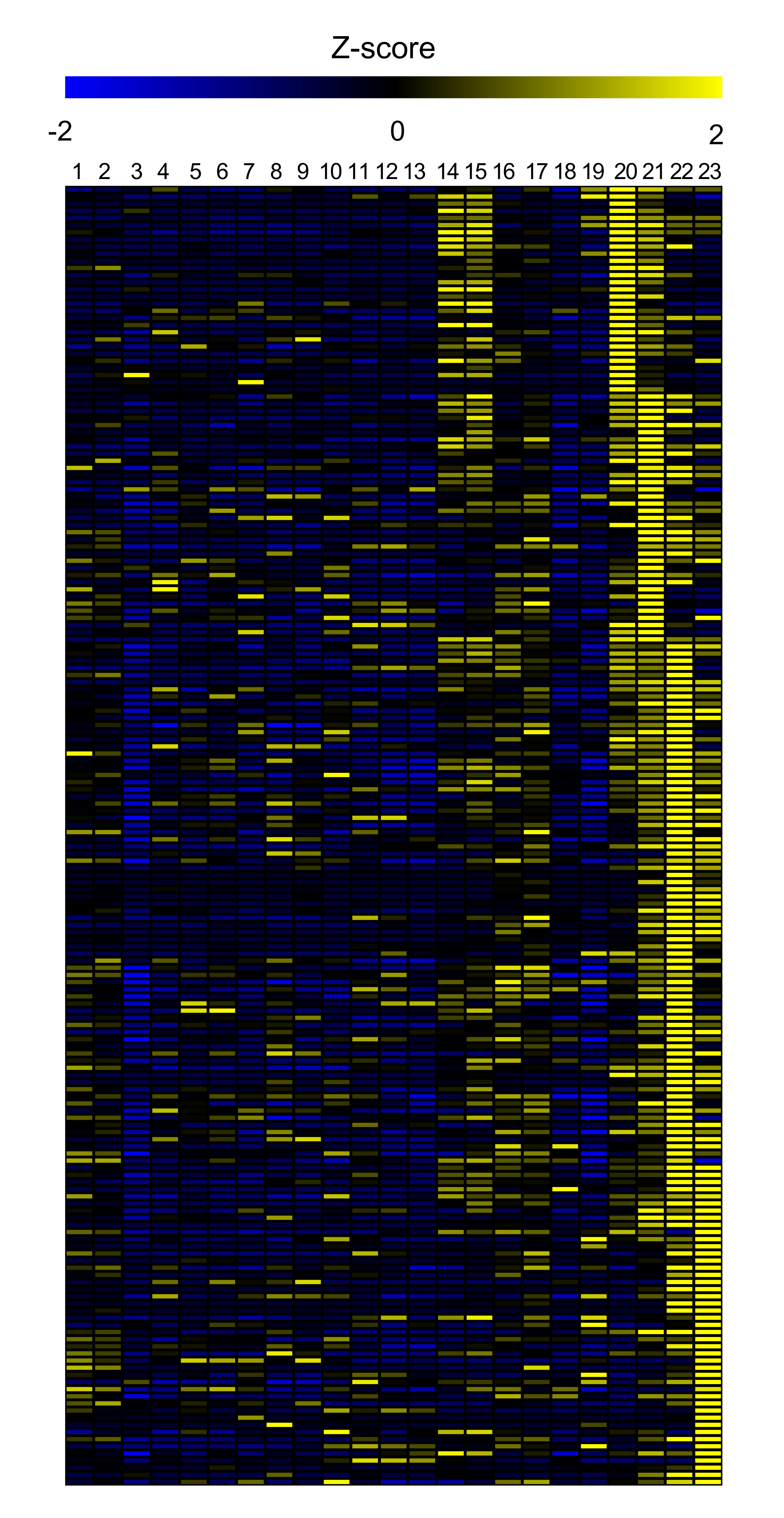

Supplement: Supplementary file 14 — Additional file 14 Figure S7. Heatmap of predominant expressed genes associated with lint percentage in cotton fiber development. The number indicated different tissues or development stages, 1: root; 2: stem; 3: leaf; 4: petal; 5: torus; 6: sepal; 7: bract; 8: anther; 9: filament; 10: pistil; 11: -3DPA ovule and fiber; 12: 0DPA ovule and fiber; 13: 1DPA ovule and fiber; 14: 3DPA ovule and fiber; 15: 5DPA ovule and fiber; 16: 10DPA ovule; 17: 15DPA ovule; 18: 20DPA ovule; 19: 25DPA ovule; 20: 10DPA fiber; 21: 15DPA fiber; 22: 20DPA fiber; 23: 25DPA fiber. (TIFF 586 kb) [file 12870_2019_2187_MOESM14_ESM.tif]

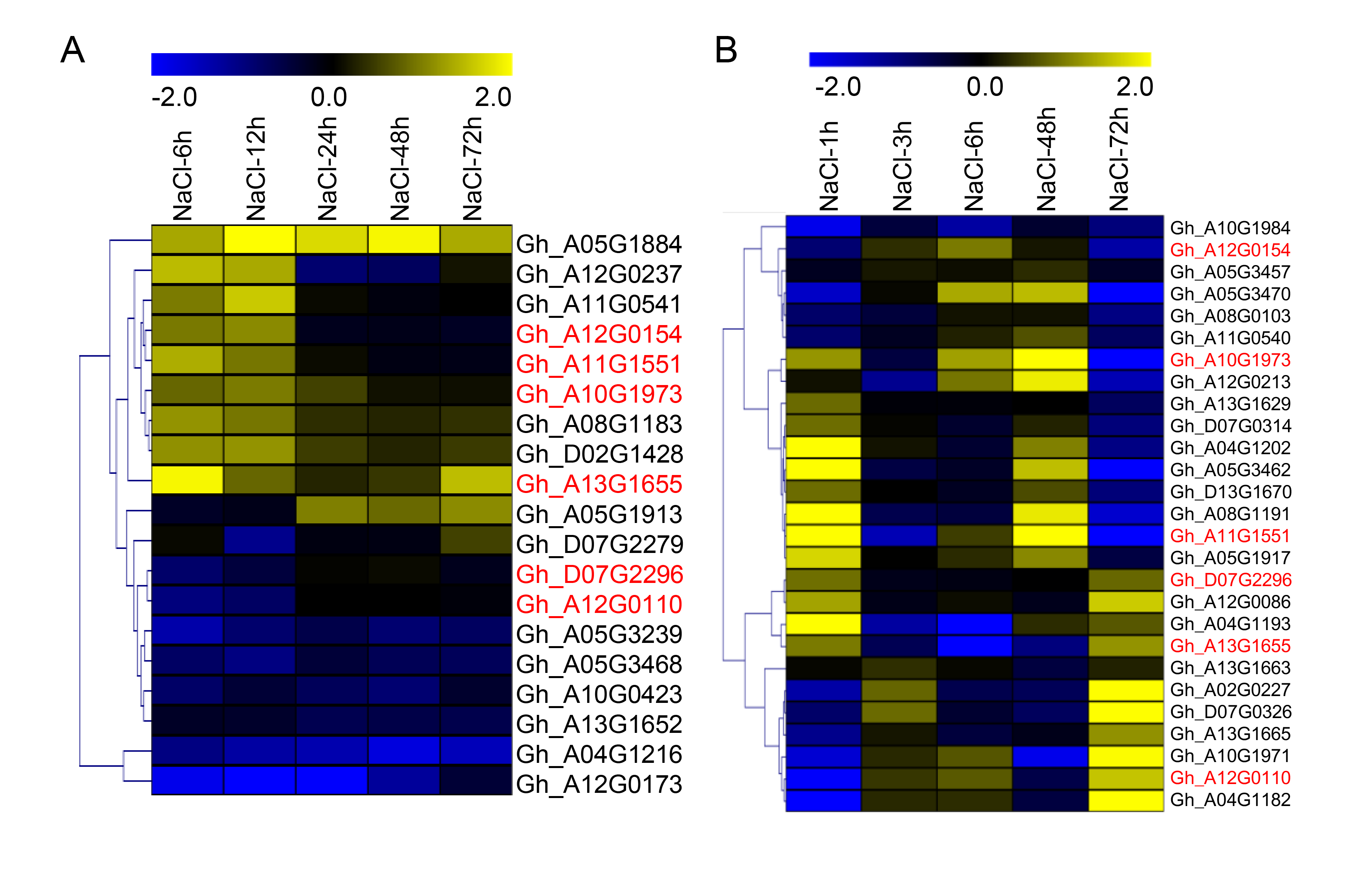

Supplement: Supplementary file 17 — Additional file 17 Figure S8. Heatmap of candidate genes related to boll number per plant under salt stress. The stress response genes located in QTLs were salt-inducible in roots (A) and leaves (B) under salt stress. The gene names marked in red represent differential expression in both roots and leaves. (TIFF 639 kb) [file 12870_2019_2187_MOESM17_ESM.tif]
